# Causal Analysis of Physiological Sleep Data Using Granger Causality and Score-Based Structure Learning

**DOI:** 10.3390/s23239455

**Published:** 2023-11-28

**Authors:** Alex Thomas, Mahesan Niranjan, Julian Legg

**Affiliations:** 1 School of Electronics and Computer Science, University of Southampton, Southampton SO17 1BJ, UK; 2 University Hospitals Southampton NHS Trust, Southampton SO16 6YD, UK

**Keywords:** causality, polysomnography, sleep medicine, structure learning

## Abstract

Understanding how the human body works during sleep and how this varies in the population is a task with significant implications for medicine. Polysomnographic studies, or sleep studies, are a common diagnostic method that produces a significant quantity of time-series sensor data. This study seeks to learn the causal structure from data from polysomnographic studies carried out on 600 adult volunteers in the United States. Two methods are used to learn the causal structure of these data: the well-established Granger causality and “DYNOTEARS”, a modern approach that uses continuous optimisation to learn dynamic Bayesian networks (DBNs). The results from the two methods are then compared. Both methods produce graphs that have a number of similarities, including the mutual causation between electrooculogram (EOG) and electroencephelogram (EEG) signals and between sleeping position and SpO2 (blood oxygen level). However, DYNOTEARS, unlike Granger causality, frequently finds a causal link to sleeping position from the other variables. Following the creation of these causal graphs, the relationship between the discovered causal structure and the characteristics of the participants is explored. It is found that there is an association between the waist size of a participant and whether a causal link is found between the electrocardiogram (ECG) measurement and the EOG and EEG measurements. It is concluded that a person’s body shape appears to impact the relationship between their heart and brain during sleep and that Granger causality and DYNOTEARS can produce differing results on real-world data.

## 1. Introduction

Sleep-related disorders not only impact quality of life but are a risk factor for serious health conditions [1]. Polysomnography, the process of recording multiple physiological signals while a patient sleeps, is considered the gold standard for diagnosing sleep-related breathing disorders [2]. Furthering our understanding of how the body functions during sleep has the potential to improve treatment; the combination of data from polysomnography and modern machine learning techniques presents an opportunity to improve this understanding.

Causal discovery, the task of identifying the relationships between variables on a causal, rather than simply correlative, level, is a current field of interest in machine learning [3]. By building a network of directed causal relations, a researcher may become able to reason about counterfactuals [4] and develop a greater understanding of a mechanism’s function. The inference of causal relationships enables interventions, which is particularly useful in a clinical context as clinicians need to intervene to treat patients. In particular, we seek to understand how causal structure varies between patients.

In this work, two different approaches to structure learning are employed: Granger causality and DYNOTEARS. The former method is a well-established technique for causal discovery, originating in the econometrics literature, while the latter was introduced in 2020 and has been gaining traction in the structure learning field.

### 1.1. Granger Causality

Granger causality (GC), or Wiener–Granger causality, was introduced by C. W. J. Granger in 1969 [5]. It is based on the principle that time series *y* causes time series *x* if the past of *y* provides information about *x* conditioned on *x*’s own past. For this work, the Multivariate Granger Causality toolbox (“MVGC toolbox”), built and maintained by Barnett and Seth [6], is used. This has over 800 citations and has been used for medical tasks including the behaviour of blood cells [7] as well as sleep [8].

In the MVGC method, time-series data are modelled as a vector autoregressive (VAR) process. For a time series U with *m* time steps and *d* variables, a *p*th order VAR model—a model in which each time step is modelled as a linear function of the previous *p* steps—may [6] be represented as

(1) $$u_{t}=\sum_{k=1}^{p}A_{k}u_{t-k}+\varepsilon_{t}.$$

$\Sigma =\mathrm{cov}(\varepsilon_{t})$ is the d×d residuals covariance matrix for this model. The model is repeatedly learned with each variable in turn excised from U to produce a series of reduced covariance matrices. For each missing variable, the pairwise-conditional Granger causality from variable *y* to *x* is the log-likelihood ratio

(2) $$F_{y\to x}=\ln\frac{|\Sigma_{\mathrm{xx}}^{\prime }|}{|\Sigma_{\mathrm{xx}}|}$$

where $\Sigma_{\mathrm{xx}}$ and $\Sigma_{\mathrm{xx}}^{\prime }$ are the residuals covariance matrices of the models with and without variable *y*, respectively. This method measures the strength of causality from one variable to another, conditioned on all the other variables in the system.

To establish the significance of the result, *p*-values are produced using an F cumulative distribution function. The null hypothesis is that there is no causality. In this study, there is considered to be a link between two variables if the *p*-value is less than 0.05, as is conventional. The results are adjusted to account for the multiple hypotheses using the method proposed by Benjamini and Yekutieli [9]. A matrix of 1s, for causality, and 0s, for no causality, is produced.

Granger causality is used in this study as it is a well-established technique for causal discoveries from time-series data. It conveys a straightforward conception of causality. Pairwise-conditional Granger causality allows each pair of variables to be conditioned on the others, avoiding confusion over variables mediating causal links.

Granger causality has been used to learn structure from polysomnographic data in previous studies. Orjuela-Cañón et al. [8] used Granger causality to study the changes brought about by a session of continuous positive air pressure (CPAP) therapy; Faes et al. [10], the impact of sleep stages; Günther et al. [11], the impact of sleep stages and apnoea; and Pizzi et al. [12] and Abdalbari et al. [13], the difference between wakefulness and sleep. These studies all involve substantially fewer subjects than this one. The objective of the Granger causality part of this study is to generate results from a larger number of subjects so that comparisons may be drawn between subjects rather than between different windows of data from the same subject.

### 1.2. Optimisation Method (DYNOTEARS)

In 2018, Zheng et al. [14] introduced the NO TEARS method of structure learning, in which an adjacency matrix is learned by minimising the loss when applied to the data, subject to a continuous acyclicity constraint and an $\ell_{1}$ penalty to enforce sparsity. DYNOTEARS [15] is an adaptation of this approach to time-series data. Instead of learning a graph with one node for each variable, a dynamic Bayesian network (DBN) is learned, in which each lag of each variable is considered a node. The NO TEARS method is adapted to learn a DBN by adding the lagged versions of the data to the loss function. To learn an inter-slice (instantaneous) adjacency matrix W and an intra-slice adjacency matrix A, again with *m* time steps and *d* variables, one generates lag matrix Y from the past *p* time steps of X and solves the following optimisation problem:

(3) $$\begin{array}{cc} \; & \min_{W,A}\frac{1}{2n}||X-\mathrm{XW}-{\mathrm{YA}||}_{F}^{2}+\lambda_{W}{\left||W|\right|}_{1}+\lambda_{A}{\left||A|\right|}_{1}\\ \; & \mathrm{subject}\;\mathrm{to}\;\mathrm{tr}(e^{W\circ W})-d=0 \end{array}$$

where n=m+1−p, $\left|\right|\cdot {\left|\right|}_{F}$ is the Froebenious norm, and $\left|\right|\cdot {\left|\right|}_{1}$ is the elementwise $\ell_{1}$ norm, which encourages the learning of a sparse model. The constraint ensures acyclicity [14]. The problem is solved using an augmented Lagrangian method due to the constraint.

DYNOTEARS is used in this study as it is a leading method in the field of structure learning by continuous optimisation. This is a field of substantial current interest in the causality community. It is of interest to compare it with Granger causality; both methods are based on learning autoregressive models from time series, but Granger causality is concerned with differences in predictive abilities between models, while DYNOTEARS involves fitting a single model to data.

DYNOTEARS has been used in previous work to learn causal graphs in domains ranging from IT service telemetry [16] to autonomous driving [17]. To the authors’ knowledge, this is the first paper in which it is applied to polysomnographic data, or to medical data in general, other than to benchmark other methods. DYNOTEARS is used here as the data are in the form of time-series signals; it is the most well-established method for using optimisation to learn structure from such data.

## 2. Materials and Methods

### 2.1. Data

The data used are from the Wisconsin Sleep Cohort Study (WSC) [18], a longitudinal study of 1500 randomly sampled Wisconsin state employees, making up a total of 2570 recordings. This study is provided by the National Sleep Research Resource (NSRR) [19]; researchers may request access to these data from the NSRR website. Written consent was provided by the participants to have their data used, and the data were pseudonymised prior to sharing.

The measurements taken during a sleep study vary throughout the dataset. The 10 variables that are most frequently included in the sleep studies in the dataset are identified; these are listed and described in Table 1 and are the variables used in this study.

These measurements were taken using a variety of instruments, and a low-pass filter was applied for most of the variables to remove high-frequency signals. The investigators in the Wisconsin study upgraded their instruments in 2009, meaning that the dataset is a combination of old 100 Hz signals and new 200 Hz signals. Details of these signals may be found in Table 2. To ensure uniformity, all time series data are resampled to 100 Hz before use in this study.

**Figure 1:**
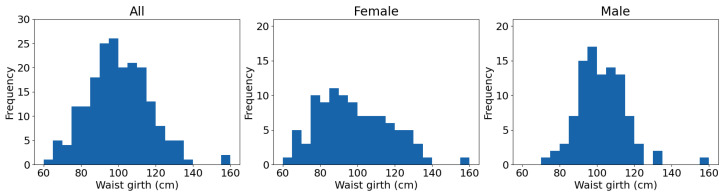
Histograms showing distribution of waist girth in test set 1. One participant omitted due to lack of waist girth measurement.

Repeat participants and sleep studies without all the chosen variables are removed, and then 200 traces are selected at random for the validation set and the two test sets. Results from the first test set are included in this paper. The demographics of this subset are given in Table 3. Distribution of waist girth, a factor that will be highlighted later, may be seen in Figure 1. One participant has been omitted from the waist girth results as there was not a waist measurement for this participant.

### 2.2. Granger Causality

#### 2.2.1. Windowing

Time series data must be covariance stationary to be modelled using vector autoregression. One would not expect this requirement to be met for a long section of polysomnographic data: sleep occurs in stages, and events occur throughout the night to affect a sleeping person’s state. Accordingly, each sleep study is split into 20-s windows. These windows are selected randomly at 10-s intervals and accepted if they are stationary; this process continues until 50 stationary windows have been chosen for each sleep study. Each data window is adjusted to have a zero mean prior to the VAR calculation.

Twenty seconds is quite a small window length; this was chosen to balance the need for as much data as possible with the requirement for stationarity. Previous studies have used windows of similar length when dealing with physiological signals [12,13].

Overlaps between windows were permitted, though only a few windows had overlaps as there was a lot of data to sample from. Overlaps were by 50%. The test for stationarity used is that the VAR model of the data, converted to first-order form, must have a coefficient matrix with a spectral radius (largest absolute eigenvalue) less than 1, as described in Lütkepohl [21].

#### 2.2.2. Model Order Selection

The model order (the maximum number of past time steps to include in the autoregression) is selected by learning the model and calculating the corrected Akaike Information Criterion (AICc) at each potential order from 1–19. The order that produces the lowest AICc is used.

### 2.3. DYNOTEARS

The data windows selected for Granger causality are used again, with the same validation/test split. Each is adjusted to have a zero mean and unit variance before applying the algorithm.

#### Selection of Hyperparameters

**Figure 2:**
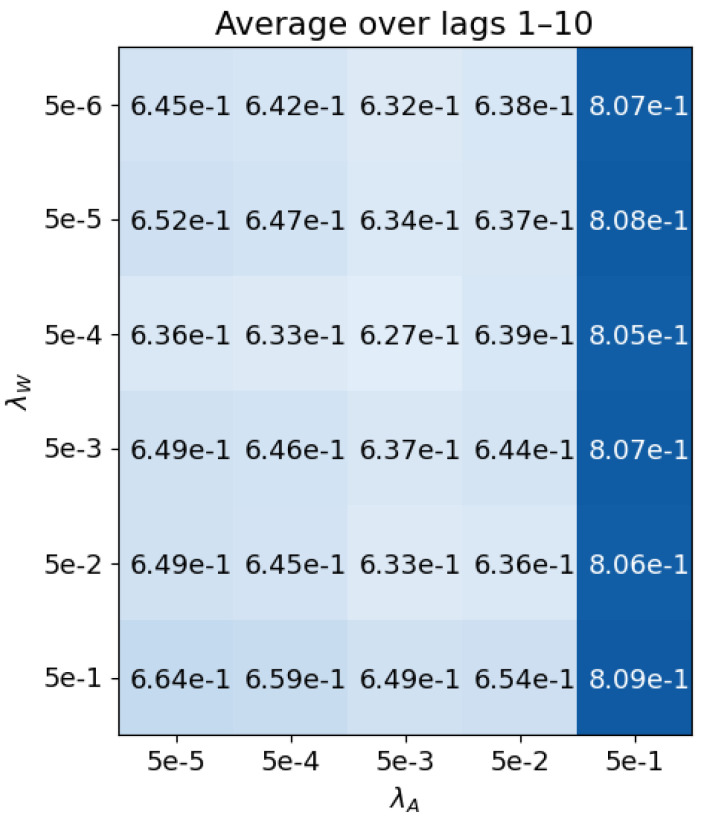
DYNOTEARS cross-validation result. Average root mean squared error (RMSE) over model orders 1–10 for each pair of hyperparameters $\lambda_{W}$ and $\lambda_{A}$. Darker colours denote a larger number.

In this case, the validation set is used to choose the two lasso coefficients $\lambda_{A}$ and $\lambda_{W}$, as well as the model order, using 10-fold cross-validation. This is carried out on all orders from 1 to 10 to find the best pair of hyperparameters for all orders. A total of 80 sleep studies are chosen at random from the validation set, with 5 randomly selected stationary windows used from each study. Figure 2 shows the average results. The chosen hyperparameters are $\lambda_{A}=0.005$ and $\lambda_{W}=0.0005$.

The model is then learned at orders 1–19, as with Granger causality, and the Akaike Information Criterion (AIC) is used to find the optimal model order.

### 2.4. Comparison with Underlying Features

A total of 210 other features provided in the dataset are compared with the causality results. Box plots are automatically drawn comparing the frequency of links between each pair of variables in the validation set with each underlying feature, and those with correlations are identified. To disregard spurious correlations, the two test sets are checked for the same correlations. A small number of these correlations appear in the results from all three datasets. By this method, the participants’ waist girth is identified as a feature of interest. Waist girth varies substantially in the population, and it is potentially significant if the functioning of the body during sleep is affected by it.

The results from applying DYNOTEARS to the test sets are subsequently checked for the waist girth correlation in order to compare them with the Granger causality results. To enable comparison between the graph of processes produced by Granger causality and the dynamic Bayesian networks produced by DYNOTEARS, a single adjacency matrix is produced in which a link is recorded between two nodes if at least one link between those nodes, at any lag, is discovered by DYNOTEARS. Links to a variable from its own past are omitted as these are assumed to exist by Granger causality (and in this case are always discovered by DYNOTEARS).

## 3. Results

### 3.1. Overall Graph Structure

**Figure 3:**
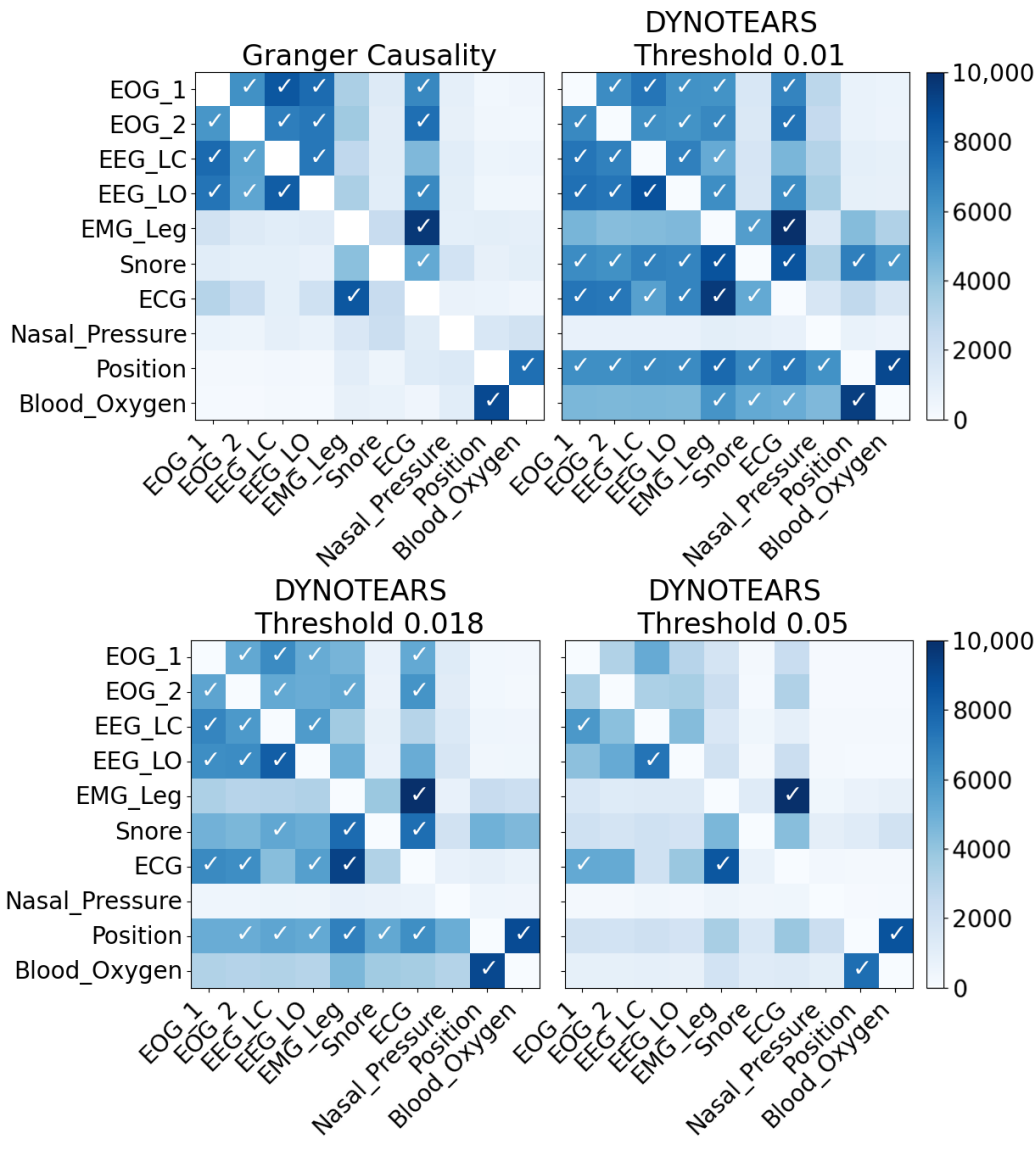
Heatmaps of Granger causality and DYNOTEARS results (test set 1). A white tick mark (✓) indicates that a link is identified in at least 50% of the 10,000 windows. Colours correspond to the scales on the right. Causal relationships are often identified between the EOG and EEG measurements and between Position and Blood_Oxygen; in addition, the ECG to EOG and EEG links are often identified.

**Figure 4:**
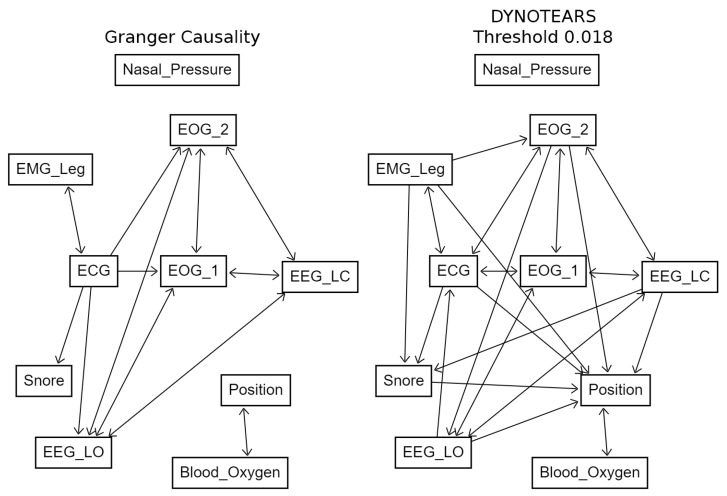
Graphs of links identified by Granger causality and DYNOTEARS (DYNOTEARS threshold 0.018) (test set 1). Threshold was chosen by manual trial and improvement to produce results similar to those from Granger causality. Graphs produced using DAGitty [22].

Figure 3 shows how frequently the 90 potential links are identified in the dataset by both methods across all the windows in the testing dataset. Those identified in at least 50% of the windows are highlighted with a tick mark. It should be noted that some of the windows fall just below the threshold for inclusion. Figure 4 depicts structure graphs, including the accepted links.

With DYNOTEARS, the relative values of the results for different links are, in general, similar to those in the Granger causality results. Which potential links are identified in each window depends on the threshold (referred to as $\tau_{A}$ and $\tau_{W}$ in the original DYNOTEARS paper, but set to the same value in the code); this is simply a value below which an entry in the adjacency matrix is set to 0.

In the DYNOTEARS paper, this is set low (to 0.01), but the arbitrary nature of it means that it is possible to select one that produces similar results to Granger causality (in this case, 0.018).

### 3.2. Impact of Waist Girth

**Figure 5:**
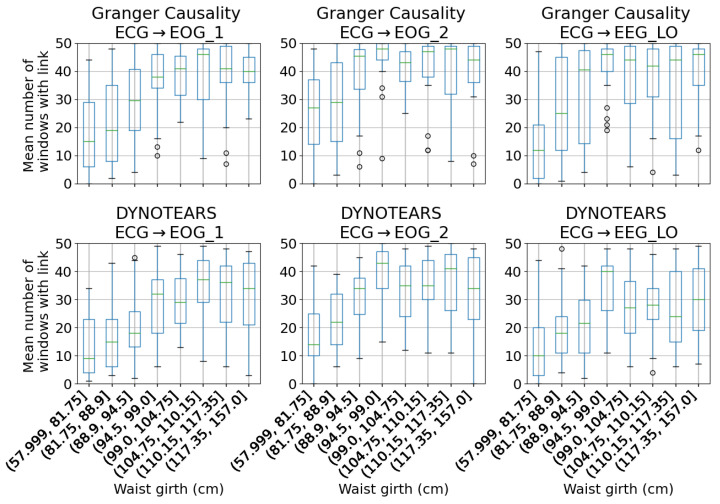
Relationship between waist girth and ECG → EOG and ECG → EEG links, using both methods (test set 1). The y-axis represents the mean number of windows, out of 50, from each sleep study in which a link is identified. Outliers (depicted as circles) are those beyond 1.5× the inter-quartile range from the lower and upper quartiles. Total 199 participants included; one participant excluded due to no waist girth measurement.

**Figure 6:**
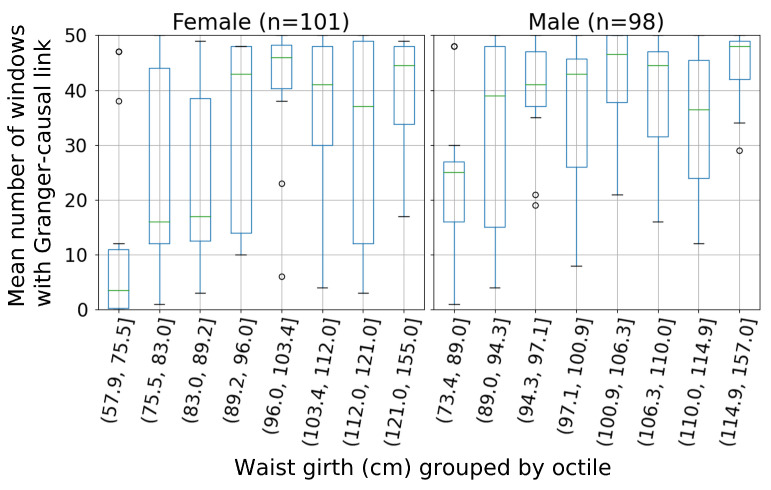
Relationship, split by sex, between waist girth and Granger causality between ECG and left occipital EEG (ECG → EEG_LO) (test set 1). Outliers (depicted as circles) are those beyond 1.5× the inter-quartile range from the lower and upper quartiles. One participant excluded due to no waist girth measurement.

When examining the results from individual sleep studies, a correlation is evident between the size of a participant’s waist and the number of windows that feature certain causal links. This is particularly notable for the links going from the electrocardiogram (ECG) trace to the electrooculogram (EOG) and electroencephalogram (EEG) traces. This is shown in Figure 5. The difference is particularly notable for participants with small waists. In Figure 6, the waist girth relationship is split by sex. The correlation is particularly pronounced among female participants with low waist girths; a contributing factor is likely that their waist size is more likely to be on the lower end (Figure 1). Most of these results have a high range over the 50 windows per participant that are tested.

These correlations were identified in the validation set and confirmed in the two test sets. This suggests that the correlation has not occurred by chance and reflects information about the functioning of the body.

The correlation is still present in the DYNOTEARS result, though it is weaker than in the Granger causality results. As usual, the choice of threshold makes a substantial difference to the results.

## 4. Discussion

This paper makes an empirical comparison of two methods of learning causal structure from data: Granger causality, developed in the econometrics literature, and the DYNOTEARS model, based on acyclicity-constrained optimisation. These data have been used for machine learning tasks by other researchers [23,24] but, to the authors’ knowledge, this is the first study that derives causal relationships from them using Granger causality or DYNOTEARS. Some links are discovered by both algorithms, with DYNOTEARS finding significantly more links that are likely to be causal. While others have attempted to infer causal relationships from physiological sensor data taken during sleep (see citations in Section 1.1), we believe that this is the first study to relate such relationships to underlying participant health.

Both methods reveal a common structure underlying the data. This structure presents several distinctive characteristics: first, these data indicate mutual causality between the EOG and EEG traces. Secondly, there appears to be a mutual causality between blood oxygen saturation levels and sleeping position. This observation is consistent with existing literature suggesting that body position can impact respiratory efficiency [25]. The identification of such causative links may assist medical professionals in making informed decisions regarding patient interventions.

On the other hand, Granger causality does not identify a pathway from sleeping position to snoring. This is in contrast with the medical literature, which finds snoring more likely among those who sleep in the supine position than those who sleep in the lateral position [26,27]. This likely suggests limitations in these results, as the correlation is well-established. DYNOTEARS, at the chosen threshold, only identifies a pathway in the opposite direction, which fits with the expected correlation but seems contrary to logical expectations.

A notable feature of the DYNOTEARS results that is not found by Granger causality is the presence of a link from the first eight variables to both position and blood oxygen level, both of which are independent of all other variables except each other in the Granger results. The reason for this difference is not clear.

The observed causal link between biopotential (EEG, EOG, and ECG) data and waist girth is intriguing, suggesting that the way the body functions during sleep varies according to body composition. The dynamic interaction between the brain and cardiac system is not well understood, although it may be associated with different phases of sleep [28]. The potential impact of body composition on this relationship could prove therapeutically helpful to clinicians if confirmed; humans have varying body shapes, and it may be that this link affects treatment. Granger causality and DYNOTEARS are useful here as they have identified a correlation between a factor and the causal link between two variables.

### 4.1. Comparison of Methods

A significant advantage of Granger causality over DYNOTEARS is its lack of hyperparameters. This means that it does not need to be repeatedly run to perform cross-validation, saving time. It also means that its results depend less on a user’s choice of whether a particular causal link is significant. At the same time, the dynamic Bayesian network structure learned by DYNOTEARS provides more detailed information about which time lag the causality is occurring at. As it produces an acyclic graph, it is possible to use its results to identify conditional independencies. Future work should investigate the specifics of these dynamic graphs.

While the definition of causality is contentious, Granger causality—a concept based on predictive ability rather than true causality—benefits from being simple to explain and based on an intuitive, logical concept. When using DYNOTEARS, one learns an adjacency matrix that minimises the loss when reconstructing the data. This may be considered a less convincing approach to learning “causal” relations than Granger’s method. Given this, it is notable that the two methods agree on many of the links in this study.

Another weakness of the NO TEARS-style approach to structure learning is that the loss function is non-convex. This means that for a particular input, there may be multiple possible adjacency matrices that correspond to a stationary point and would be considered solutions; the algorithm will only return one of these. However, in the empirical tests published in the original NO TEARS paper, the obtained solution is often close to the ground truth. The authors suggest that this is evidence that non-convexity is a minor issue in practice [14]. The paper that introduced DYNOTEARS does not make a similar statement, but the method is shown to be competitive with alternative algorithms [15]. A comparison with Granger causality is not made.

Both methods can identify how physiological factors affect the functioning of a person’s body during sleep. Further work should investigate how this relates to other factors that were not used in this study.

### 4.2. Limitations

Not all data may be modelled meaningfully using least-squares linear autoregressive models; other, more complex models should be used to identify non-linear relationships.

EMG signals often have a high frequency. The sampling frequency is set by the instruments used by the Wisconsin study and may be too low to identify some of these high frequencies; therefore, there may be aliasing in the data. This may limit the usefulness of the causality calculations.

The maximum lag order of 19, while enough to identify a significant number of relationships using both methods, is short. This maximum order may well miss longer-term causal relationships.

This study is limited by its inability to account for other factors for which there are no data available. It is all but certain that causal sufficiency, the assumption that all relevant variables are measured, is not achieved here. This limits the conclusions that can be drawn due to the possible confounding of links by unobserved factors.

## 5. Conclusions

Understanding the functioning of the body during sleep is a problem with clinical relevance, and causal discovery from time-series data may assist with this. This paper uses two methods to learn the causal structure from time-series polysomnographic data: Granger causality and the continuous optimisation method DYNOTEARS. The two methods produce structures that are similar in some aspects but vary in others; in particular, features causing body position are more frequently identified by DYNOTEARS than by Granger causality. Finally, a correlation exists between participants’ waist girth and the frequency of identification of the ECG → EOG and ECG → EEG links.

## Figures and Tables

**Table 1:** Descriptions of measurements used.

Abbreviation	Name in Dataset	Definition
EOG_1	E1	Left electrooculogram (EOG)
EOG_2	E2	Right electrooculogram (EOG)
EEG_LC	C3_M2	Left central electroencephalogram (EEG)
EEG_LO	O1_M2	Left occipital electroencephalogram (EEG)
EMG_Leg	lleg_r	Linked left and right leg electromyogram (EMG)
Snore	snore	Snore
ECG	ECG	Electrocardiogram (ECG)
Nasal_Pressure	nas_pres	Nasal pressure
Position	position	Position
Blood_Oxygen	spo2	Blood oxygen

Descriptions adapted from National Sleep Research Resource [20]. Names used in the “Abbreviation” column will be used in this paper for brevity and clarity. The “Name in Dataset” column includes the names as used in the original dataset.

**Table 2:** Settings for signals used. Sampling rate (prior to resampling) and low-pass filter used on each type of signal. Adapted from National Sleep Research Resource [20].

	2000–2009		Post–2009	
**Variable**	**Sampling Rate (Hz)**	**Hardware Filter (Hz)**	**Sampling Rate (Hz)**	**Hardware Filter (Hz)**
EOG_1	100	Low Pass 30	200	Low Pass 35
EOG_2	100	Low Pass 30	200	Low Pass 35
EEG_LC	100	Low Pass 30	200	Low Pass 35
EEG_LO	100	Low Pass 30	200	Low Pass 35
EMG_Leg	100	Low Pass 30	200	Low Pass 70
Snore	100	Low Pass 30	200	Low Pass 70
ECG	100	Low Pass 30	200	Low Pass 35
Nasal_Pressure	100	Low Pass 30	200	Low Pass 15
Position	100	-	200	-
Blood_Oxygen	100	-	200	-

**Table 3:** Demographics of participants in test set 1.

Variable	Category	Frequency	Percent
Sex	Male	98	49.0%
	Female	102	51.0%
Age	30 < x ≤ 40	2	1.0%
	40 < x ≤ 50	31	15.5%
	50 < x ≤ 60	83	41.5%
	60 < x ≤ 70	71	35.5%
	70 < x ≤ 80	13	6.5%
BMI	10 < x ≤ 20	7	3.5%
	20 < x ≤ 30	93	46.5%
	30 < x ≤ 40	73	36.5%
	40 < x ≤ 50	17	8.5%
	50 < x ≤ 60	9	4.5%
	60 < x ≤ 70	1	0.5%
Race	Asian	1	0.5%
	Black	3	1.5%
	Hispanic	1	0.5%
	Native American	0	0.0%
	White	195	97.5%

## Data Availability

Access to the Wisconsin Sleep Cohort data is restricted due to the sensitivity of medical information. Access may be requested at https://sleepdata.org/datasets/wsc accessed on 1 December 2021.

## Abbreviations

In addition to the polysomnography variable names described in Table 1, the following abbreviations are used in this manuscript:

AIC	Akaike Information Criterion
AICc	Corrected Akaike Information Criterion
CPAP	Continuous positive air pressure
DBN	Dynamic Bayesian network
(MV)GC	(Multivariate) Granger causality
NSRR	National Sleep Research Resource
VAR	Vector autoregression
WSC	Wisconsin Sleep Cohort
